# Supplementary material for: Improving tuberculosis case detection through contact risk stratification by Xpert MTB/RIF Ultra and spatial parameters: Evaluation of an innovative active case finding strategy in Mozambique (Xpatial-TB)
Source: PLOS Glob Public Health. 2024 Feb 9;4(2):e0002789. doi: 10.1371/journal.pgph.0002789 (PMC10857722; doi:10.1371/journal.pgph.0002789)
Supplement: S1 Text — Table A. Screening radii calculations, Fig A. Maps. (DOCX) [file pgph.0002789.s001.docx]

Improving case detection through TB contact risk stratification by Xpert MTB/RIF Ultra and spatial parameters. Evaluation of an innovative ACF strategy in Mozambique.

**Supporting information**

**S1.**  **Xpatial-TB procedures additional details**

*Passive Case Finding (PCF) activities*

Index cases (ICs) were passively identified following National guidelines. In 2018, the National TB program (NTP) was working in the district through a network of 10 peripheral healthcare centres (Calanga, Chibubutzo, Ilha Josina, Malavela, Maluana, Maragra, Munguine, Palmeiras, Taninga, 3 de Fevereiro), and two hospitals (Manhiça District Hospital, MDH, and Xinavane Rural Hospital, XRH) (Map S1). Only health facilities associated with both hospitals had the infrastructure for TB diagnosis through molecular testing.

As part of the TB diagnostic workup, presumptive pulmonary TB cases, attending any of the health facilities were asked to provide sputum for testing. Samples were directed to the laboratories associated with both, MDH or XRH in a daily basis.

Patients were managed according to the National Tuberculosis Programme (NTP) guidelines and initiated on treatment shortly after TB confirmation or high diagnostic suspicion (clinically diagnosed cases). Registration in the NTP official books and treatment (modified DOTS on a weekly basis) occur at the two hospitals but also at the 10-peripheral health centres of the district. National protocols recommend a follow up clinical visit after two weeks of treatment and monthly until the end of treatment (1).

The study coordinator and the Xpatial-TB field teams worked together with the NTP staff in each of the health care units in order to identify and recruit all new and relapse cases initiating treatment in the district.

*Eligible population: Screening radii for community contact identification*

The screening radii for active-case finding (ACF) was calculated based on the household density of the neighbourhood where the IC lived. Population density was calculated using information from 261 neighbourhoods, which ranged from 0.07 and 2161.6 per square km. Three levels of household density were established through statistical simulations of predefined radius, to ensure an average of 5 households per strata per IC, and a similar number of individuals (assuming 5 individuals per household). The three groups and corresponding radii can be observed in Table S1.

**Table A. Screening radii calculations**

| Group | Household density (n/km^2^) | Screening Radius | Mean number of households using predefined radii | Mean of individuals that would be screened |
| --- | --- | --- | --- | --- |
| *Low density* | <100 | 120 | 4.12 | 20.6 |
| *Medium density* | 100-450 | 70 | 7.59 | 37.5 |
| *High density* | >450 | 40 | 6.69 | 33.45 |

These groups were identified so that (i) there would be a minimum of 20 individuals screened per radius and (ii) study resources were available to limit a maximum radius and maximum number of people screened per IC.

*Sample collection*

*PCF:* Raw samples were collected at the health units and sent to the MDH or XRH (depending on proximity) for Xpert Ultra testing. Study samples were stored daily at the BSL3 laboratory of the Manhiça Health Research Center (CISM).

ACF: All participants who fulfilled testing criteria were requested to provide one spot sputum specimen in the field with a minimum volume of 2 ml. Sputum induction was used at participants’ household for those unable to provide spontaneous sputum, by administering one inhalation of salbutamol (100 micrograms) followed by 15 minutes of nebulization with hypertonic saline solution (5%) with portable nebulizers (OMRON MicroAIR U22).

*Laboratory methods*

Xpert Ultra MTB/RIF (Cepheid. Sunnyvale. CA. USA. Hereinafter referred to as Xpert Ultra) is a real-time PCR which can identify *Mycobacterium tuberculosis complex* (MTBC) and markers of resistance to rifampicin(1). It provides semi quantitative results depending on the quantity of genetic material detected that have shown good correlation with other estimates of bacillary load(2). Respiratory specimens were tested according to the manufacturing instructions. Invalid results were repeated. When rifampicin resistance was detected by Xpert Ultra, liquid culture (BACTEC Mycobacterium Growth Indicator Tubes (MGIT)), and phenotypic drug susceptibility tests were performed (Becton Dickinson Microbiology System. USA), as per national guideline.

*
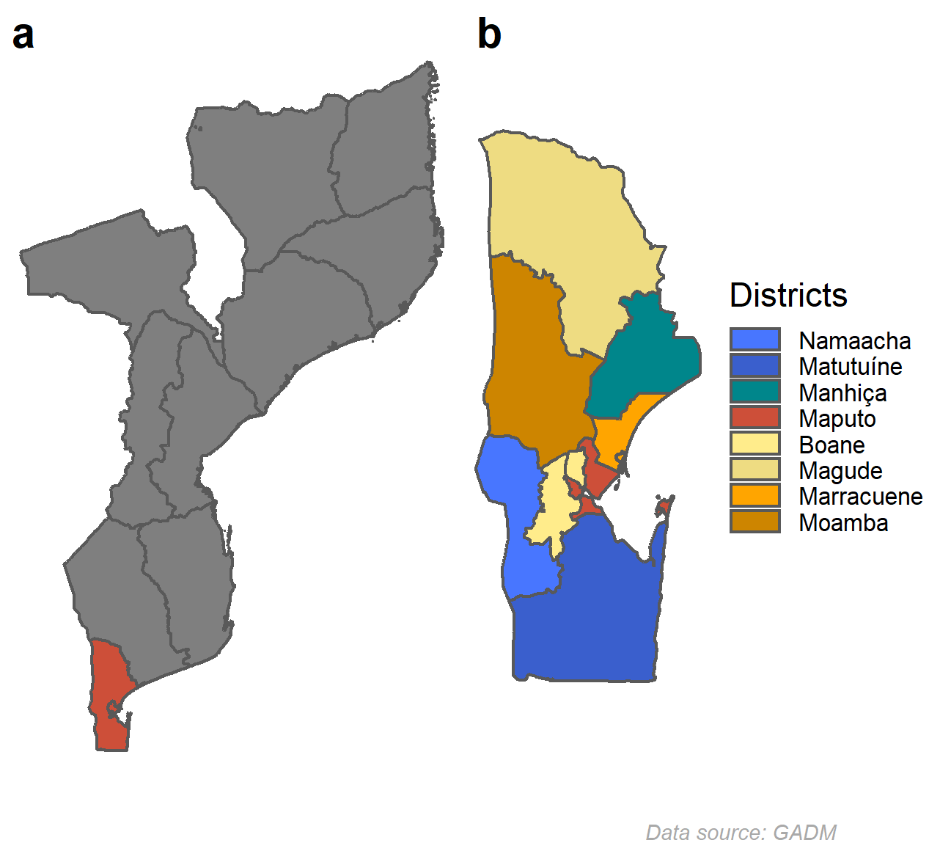
*

**Fig A. Maps. a) Maputo Province in the map of Mozambique. b) Districts in Maputo Province. Intervention area: Manhica district (green colour) and control area (South of Maputo province, which includes the districts of Namaacha and Matutuine, in two tons of blue colour)**

Two districts in Maputo province, Namaacha and Matutuine, were chosen as a control area for the CITS analysis based on the following: i) Similar to Manhiça, they are rural districts close to the capital of the province; ii) they did not use molecular tests for TB diagnosis during 2018; iii) they do not border Manhiça district in order to avoid case transfer; iii) the combined population of both districts is similar to Manhiça´s population estimates.

*Maps has been created in R version 4.1.1 (2021-08-10). Basemap shape file (country and district boundaries) has been provided under an open license (CC-BY) by GADM, v. 4,.1. (https://gadm.org/data.html)*

**References**

1. Global Laboratory Initiative. Planning for country transition to Xpert ® MTB/RIF Ultra Cartridges [Internet]. Geneva; 2017. Available from: http://www.stoptb.org/wg/GLI/assets/documents/GLI_ultra.pdf

2. Beynon F, Theron G, Respeito D, Mambuque E, Saavedra B, Bulo H, et al. Correlation of Xpert MTB/RIF with measures to assess Mycobacterium tuberculosis bacillary burden in high HIV burden areas of Southern Africa. Sci Rep [Internet]. 2018 [cited 2018 Jun 27];8(1). Available from: https://www.ncbi.nlm.nih.gov/pmc/articles/PMC5980110/pdf/41598_2018_Article_23066.pdf
