# Supplementary material for: Improving tuberculosis case detection through contact risk stratification by Xpert MTB/RIF Ultra and spatial parameters: Evaluation of an innovative active case finding strategy in Mozambique (Xpatial-TB)
Source: PLOS Glob Public Health. 2024 Feb 9;4(2):e0002789. doi: 10.1371/journal.pgph.0002789 (PMC10857722; doi:10.1371/journal.pgph.0002789)
Supplement: S2 Text — (DOCX) [file pgph.0002789.s002.docx]

**S2. Interrupted time-series for single and multiple-group comparison**

ITS and CITS were undertook based on the following:

1) This design is generally applied to interventions introduced at a known point in time.

2) We collected data at regular intervals over time

3) By modelling trends, we not only compared the intervention outcomes before & after, but also attempted to avoid misleading conclusions due to random or secular trends.

A. Interrupted time-series (ITS) model 1

Y_t_=β_0_ + β_1_T_t_ + β_2_X_t_ + β_3_X_t_T_t_

*Terms*

Y_t :_ is the outcome measure along time (Notification Rate= cases/100,000 population)

T_t:_ is the quarter time counter

X_t:_ indicates pre- and post-intervention periods (dummy variable; pre-intervention (before 2018)=0/intervention(2018)=1)

X_t_T_t:_ interaction term

*Coefficients*

**β_0_** (intercept) pre-intervention period;

**β_1_** pre-intervention slope

**β_2_**  post-intervention step change

**β_3_** difference pre-post intervention trends

B. Control interrupted time-series (CITS) model 2. Comparisons Manhiça/South Maputo

The model measures differences in post-intervention step-changes and trends in comparison with the control group .

Y_t_=β_0_+β_1_T_t_+β_2_X_t_+β_3_X_t_T_t_+β_4_Z+β_5_ZT_t_+β_6_ZX_t_+β_6_ZX_t_T_t_


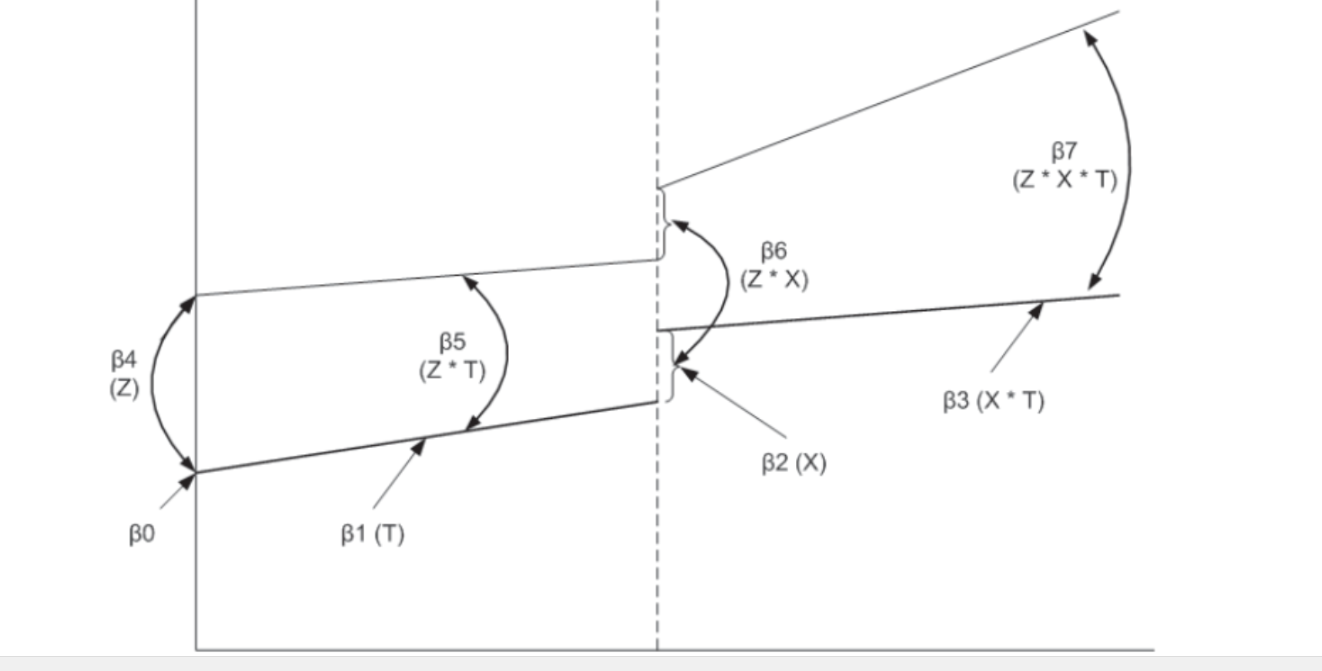


Visual description of model terms. Extracted from *Conducting interrupted time-series analysis for*

*single- and multiple-group comparisons* (1)

Yt: is the outcome measure along time (Incidence Rate= cases/100,000 population);

Tt: is the quarter time counter;

Xt :indicates pre- and post-intervention periods (dummy variable; pre-intervention=0/post-intervention=1)

Z: denotes the intervention cohort (dummy variable; Manhiça=1/South Maputo=0)

ZTt, ZXt, and ZXtTt are interaction terms.

*Coefficients*

**β_0_ to β_3_** relate to the control group as follows: β_0_ (intercept) pre-intervention period; β_1_ pre-intervention slope; β_2_  post-intervention step change; β_3_ difference pre-post intervention trends

**β_4_ to β_7_** represent differences between the control and intervention districts: β_4_ difference in baseline intercepts; β_5_ difference in pre-intervention trends; β_6_ difference in post-intervention step changes; β_7_ post-intervention trend difference.

C. Databases

Since 2016, the NTP counts with digital registers of TB notifications (SIS-MA) as aggregated data reports that are subjected to data cleaning and duplicate removal. This information derives from individual TB case reporting obtained from the data collected on paper-based registries (official NTP books). Therefore, the number of cases for 2015, 2016, 2017 and 2018 was obtained from quarterly aggregated information provided by the national surveillance system (official reports). Population data was provided by the national institute of statistics for the control areas, and by the HDSS .

Data derived from ACF activities were collected using tablets and managed electronically in a secure REDCap database (Vanderbilt University, Nashville, TN, USA; 2016)

**Reference**

1. Linden A. Conducting interrupted time-series analysis for single- and multiple-group comparisons. Stata J. 2015;15(2):480–500.
