# Supplementary material for: Improving tuberculosis case detection through contact risk stratification by Xpert MTB/RIF Ultra and spatial parameters: Evaluation of an innovative active case finding strategy in Mozambique (Xpatial-TB)
Source: PLOS Glob Public Health. 2024 Feb 9;4(2):e0002789. doi: 10.1371/journal.pgph.0002789 (PMC10857722; doi:10.1371/journal.pgph.0002789)
Supplement: S1 Table — (DOCX) [file pgph.0002789.s003.docx]

**S1 Table. Absolute numbers to calculate NNS (number needed to screen)**

|  | HCs^1^ | CCs^2^ | total |
| --- | --- | --- | --- |
| All screened | 3165 | 4730 | 7895 |
| TB cases | 58 | 31 | 89 |
| NNS^3^ | 54.6 | 152.6 | 88.7 |
|  |  |  |  |
| All screened | 3165 | 4730 | 7895 |
| Lab-confirmed | 27 | 20 | 47 |
| NNS^4^ | 117.2 | 236.5 | 168.0 |
|  |  |  |  |
| All screened | 3165 | 4730 | 7895 |
| Symptomatic cases | 55 | 24 | 79 |
| NNS^5^ | 57.5 | 197.1 | 99.9 |
|  |  |  |  |
| All screened | 3165 | 4730 | 7895 |
| Asymptomatic TB/HIV cases | 3 | 7 | 10 |
| NNS^6^ | 1055.0 | 675.7 | 789.5 |
|  |  |  |  |
| All screened <12 yo | 1612 | 2204 | 3816 |
| Paediatric cases | 18 | 2 | 20 |
| NNS^7^ | 89.6 | 1102.0 | 190.8 |
|  |  |  |  |
| All screened | 3165 | 4730 | 7895 |
| women TB cases | 28 | 20 | 48 |
| NNS^8^ | 113.0 | 236.5 | 164.5 |
|  |  |  |  |
| All screened | 3165 | 4730 | 7895 |
| men TB cases | 30 | 11 | 41 |
| NNS^9^ | 105.5 | 430.0 | 192.6 |
|  |  |  |  |
| women screened | 1525 | 1939 | 3464 |
| women TB cases | 28 | 20 | 48 |
| NNS^10^ | 54.5 | 97.0 | 72.2 |
|  |  |  |  |
| men screened | 1640 | 2791 | 4431 |
| men TB cases | 30 | 11 | 41 |
| NNS^11^ | 54.7 | 253.7 | 108.1 |
|  |  |  |  |

Footnote: 1) HCs: Household contacts; 2) CCs: community close contacts; 3) NNS: number needed to screen to find a TB cases in the overall study population; 4) number needed to screen to find a laboratory—confirmed cases; 5) NNS to find a symptomatic patient; 6) number needed to screen to find an asymptomatic coinfected TB/HIV patient; 7) NNS to find a patient < 12 years old ; 8 ) NNS to find a women with TB; 9) NNS to find a men with TB ; 10) women needed to screen to find a TB case; 11) men needed to screen to find a TB case
