# Supplementary material for: Improving tuberculosis case detection through contact risk stratification by Xpert MTB/RIF Ultra and spatial parameters: Evaluation of an innovative active case finding strategy in Mozambique (Xpatial-TB)
Source: PLOS Glob Public Health. 2024 Feb 9;4(2):e0002789. doi: 10.1371/journal.pgph.0002789 (PMC10857722; doi:10.1371/journal.pgph.0002789)
Supplement: S2 Table — (DOCX) [file pgph.0002789.s004.docx]

**S2 Table. Characteristics of screened household and community contact**

|  | **HCs^1^ (%)^2^** | **CCs^3^ (%)** | **Total (%)** | p-value^4^ |
| --- | --- | --- | --- | --- |
| **Identified** | 4,394 | 6,373 | 10,767 |  |
| **Screened (% from identified)** | 3,165 (72.0) | 4,730 (74.2) | 7,895 (73.3) |  |
| **HIV Status** |  |  |  | <0.001 |
| *Positive* | 344 (10.9) | 357 (7.5) | 701 (8.9) |  |
| *Negative* | 2,756 (87.1) | 4,319 (91.3) | 7,075 (89.6) |  |
| *Invalid/Undetermined* | 1 (0.03) | 8 (0.2) | 9 (0.1) |  |
| *Refuse test* | 43 (1.4) | 46 (1.0) | 89 (1.1) |  |
| *missing* | 21 (0.7) | 0 | 21 (0.3) |  |
| **Sex** |  |  |  | 0.25 |
| *Women* | 1738 (54.9) | 2660 (56.2) | 4398 (55.7) |  |
| *Men* | 1427 (45.1) | 2070 (43.7) | 3497 (44.3) |  |
| **Median age [IQR]** | 14.6 [7.78;30.3] | 16.4 [8.25;33.3] | 15.5 [8.0;32.4] | <0.001 |
| *Median age women* |  |  | 15.1 [7.9;31.1] | 0.08 |
| *Median age men* |  |  | 15.8 [8.1;33.2] |  |
| **Age group** |  |  |  | < 0.001 |
| *<5* | 470 (14.9) | 679 (14.4) | 1149 (14.5) |  |
| *5-15* | 1149 (36.5) | 1525 (32.2) | 2674 (33.9) |  |
| *15-35* | 844 (26.8) | 1418 (30.0) | 2262 (28.6) |  |
| *35-55* | 392 (12.4) | 709 (15.0) | 1101 (13.9) |  |
| *55-75* | 215 (6.8) | 322 (6.8) | 537 (6.8) |  |
| *+75* | 75 (2.4) | 76 (1.6) | 151 (1.9) |  |
| *missing* | 20 (0.6) | 1 (0.02) | 21 (0.3) |  |
| **Follow-up unit** |  |  |  |  |
| ***Manhiça (HD & CS)*** | 1027 (32.4) | 1743 (36.8) | 2770 (35) |  |
| ***First degree relatives^5^*** | 1537 (48.5) | na | na |  |

**Footnote: 1) HCs: Household contacts; 2) proportions show column percentages; 3) CCs: Community close contacts; 4) Chi-square or Fisher-exact test calculated for categorical variables and Kruscal-Wallis test to compare median age among groups; 5) First degree’s relatives include: parent, step-parent, daughter, son, siblings or partner.**
