# Supplementary material for: Improving tuberculosis case detection through contact risk stratification by Xpert MTB/RIF Ultra and spatial parameters: Evaluation of an innovative active case finding strategy in Mozambique (Xpatial-TB)
Source: PLOS Glob Public Health. 2024 Feb 9;4(2):e0002789. doi: 10.1371/journal.pgph.0002789 (PMC10857722; doi:10.1371/journal.pgph.0002789)

**S1 Fig. Visualization of interrupted time series analysis for Manhiça district**

**Fig A. Visualization of interrupted time series analysis for Manhiça district (notification of total number of cases)** **
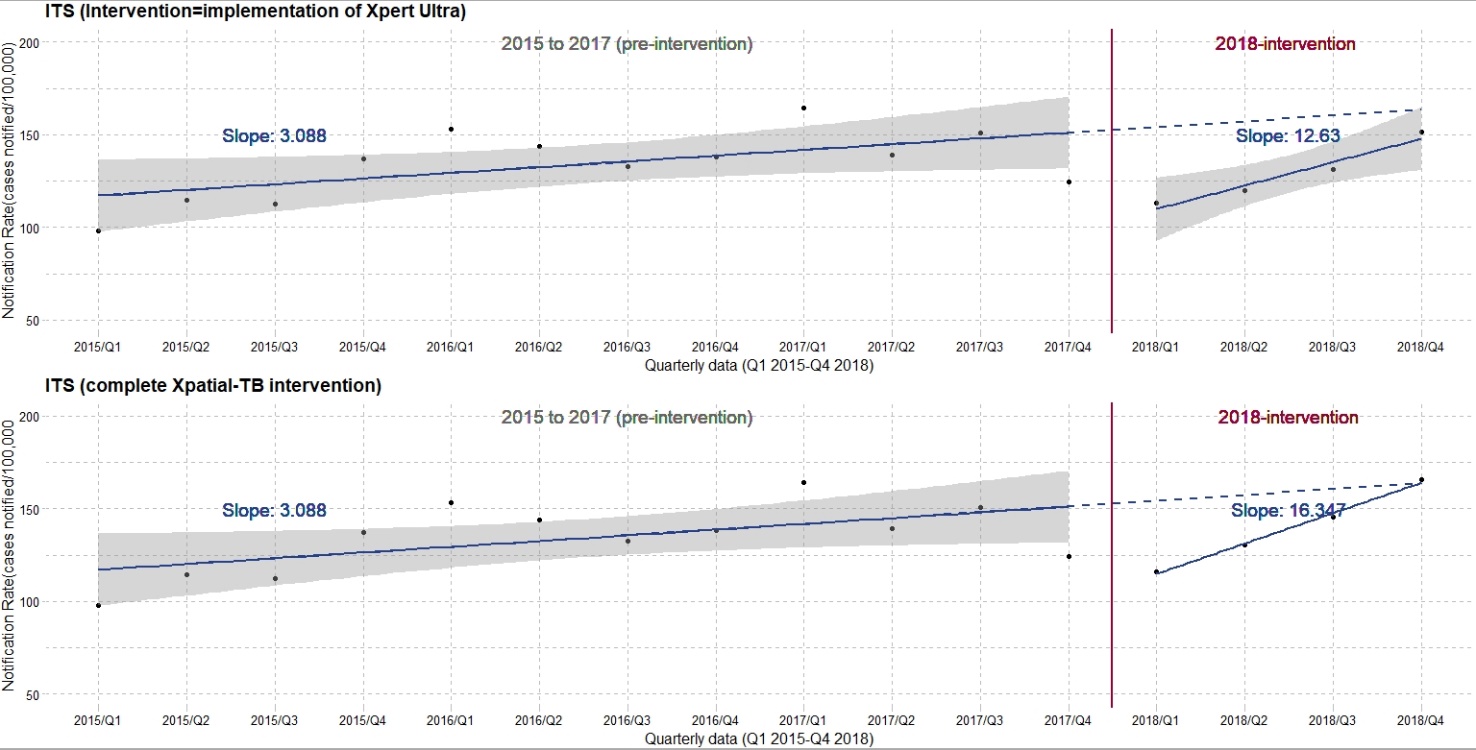
****Fig B. Visualization of interrupted time series analysis for Manhiça district (notification of microbiologically confirmed cases)**


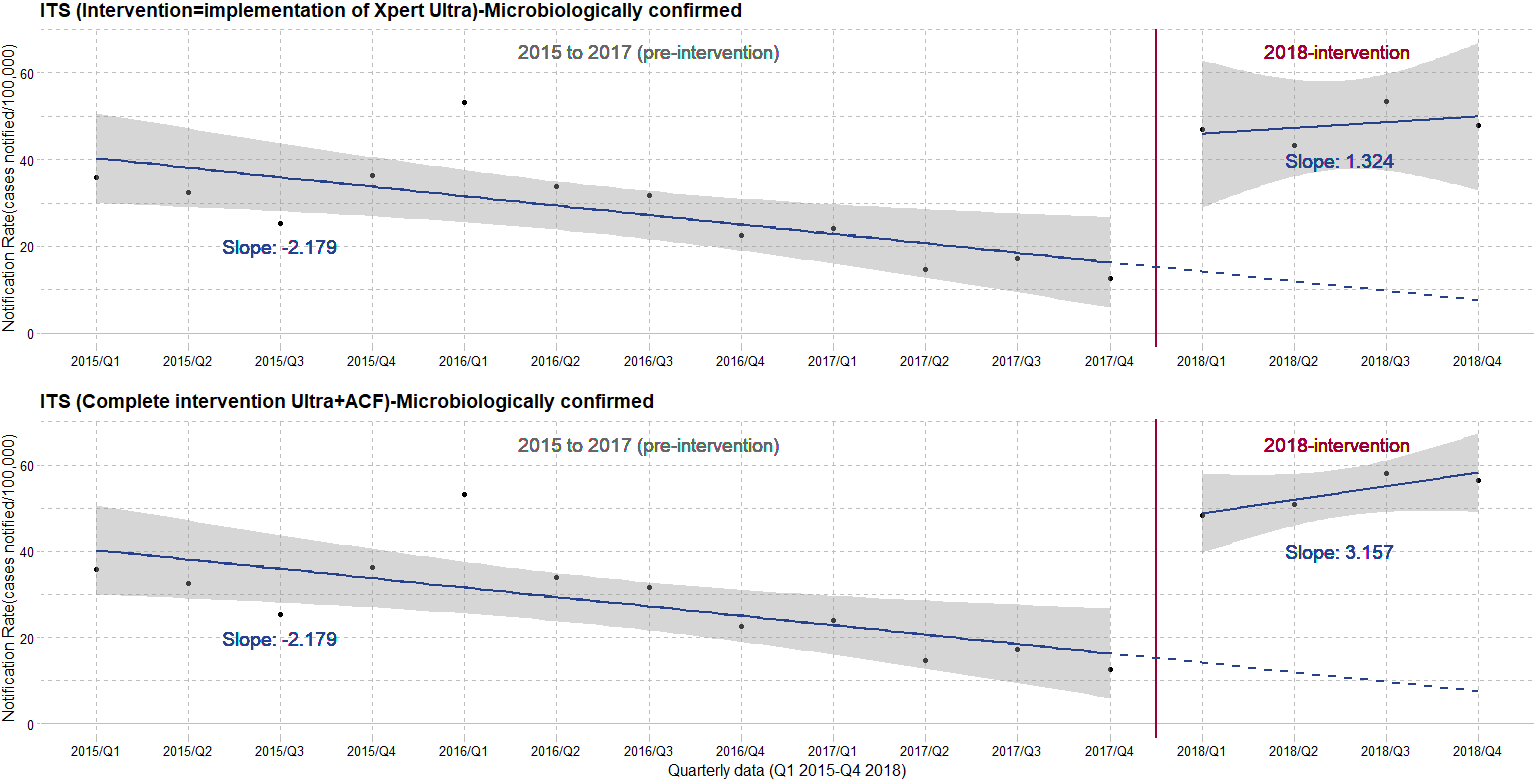

Supplement: S1 Fig — Fig A. Visualization of interrupted time series analysis for Manhiça district (notification of total number of cases), Fig B. Visualization of interrupted time series analysis for Manhiça district (notification of microbiologically confirmed cases). (DOCX) [file pgph.0002789.s005.docx]
