# Supplementary material for: Improving tuberculosis case detection through contact risk stratification by Xpert MTB/RIF Ultra and spatial parameters: Evaluation of an innovative active case finding strategy in Mozambique (Xpatial-TB)
Source: PLOS Glob Public Health. 2024 Feb 9;4(2):e0002789. doi: 10.1371/journal.pgph.0002789 (PMC10857722; doi:10.1371/journal.pgph.0002789)
Supplement: Attachment — Submitted filename: Response_to_reviewers_July2023_PGPHD_final.pdf [file pgph.0002789.s008.pdf]

cism
centro de
investigação
em saúde de
manhiça

Manhiça, 17th July 2023

Dear Dr Raquel Muñiz-Salazar,

Thank you for the opportunity to revise and resubmit the manuscript entitled ***"Improving tuberculosis case detection through contact risk stratification by Xpert MTB/RIF Ultra and spatial parameters: Evaluation of an innovative active case finding strategy in Mozambique (Xpatial-TB)"*** which we re-submit for publication in *PLOS Global Public Health* after receiving the feedback from the reviewers and editorial team. We have carefully reviewed the new comments and effected changes to the manuscript where appropriate. Below, please find our itemized responses.

To expedite the review of this revised manuscript, we have uploaded two new versions of the documents; i) *'Manuscript_July2023'* ('clean version) that includes the unmarked version of the revised manuscript, and ii) '*Revised_Manuscript_Track_Changes_July2023'*, as requested, to visualize where changes have been made without difficulties.

Additional Editor Comments

The authors have taken into account most of the feedback from the previous review. However, one major issue is that the conclusion statement in the abstract lacks evidence from the data presented. According to the abstract, there was a rise in laboratory-confirmed TB cases in the intervention district as compared to the control area, as per the CITS model. However, Table 4 does not reveal any significant variation in the step change or intervention trends of the two areas. Rephrase this statement to ensure it corresponds with the outcomes without changing its meaning, adding any new information, new sentences or paragraphs, and removing any important information from the text. Please use the same formality style for the rewrite as the one used in the original text.

Response: Thank you for bringing this to our attention. Indeed, this finding is extensively discussed in the fourth paragraph of our discussion section. We emphasize the change in bacteriological confirmation, as evidenced by Figure 4 (see below), although the analysis did

not reveal a statistically significant difference. We acknowledge that this discrepancy could be attributed to the limited data points considered during the intervention period. Thus we hypothesize that a more comprehensive dataset might provide further insights into the observed changes.

Nevertheless, we do agree with your comment and we have rephrased this sentence in the abstract accordingly. Now it reads: *During the intervention period, a reversal of the previous negative trend in lab-confirmed case notifications was observed in the Manhiça district. However, the CITS model did not show any statistically significant difference when compared to the control area*

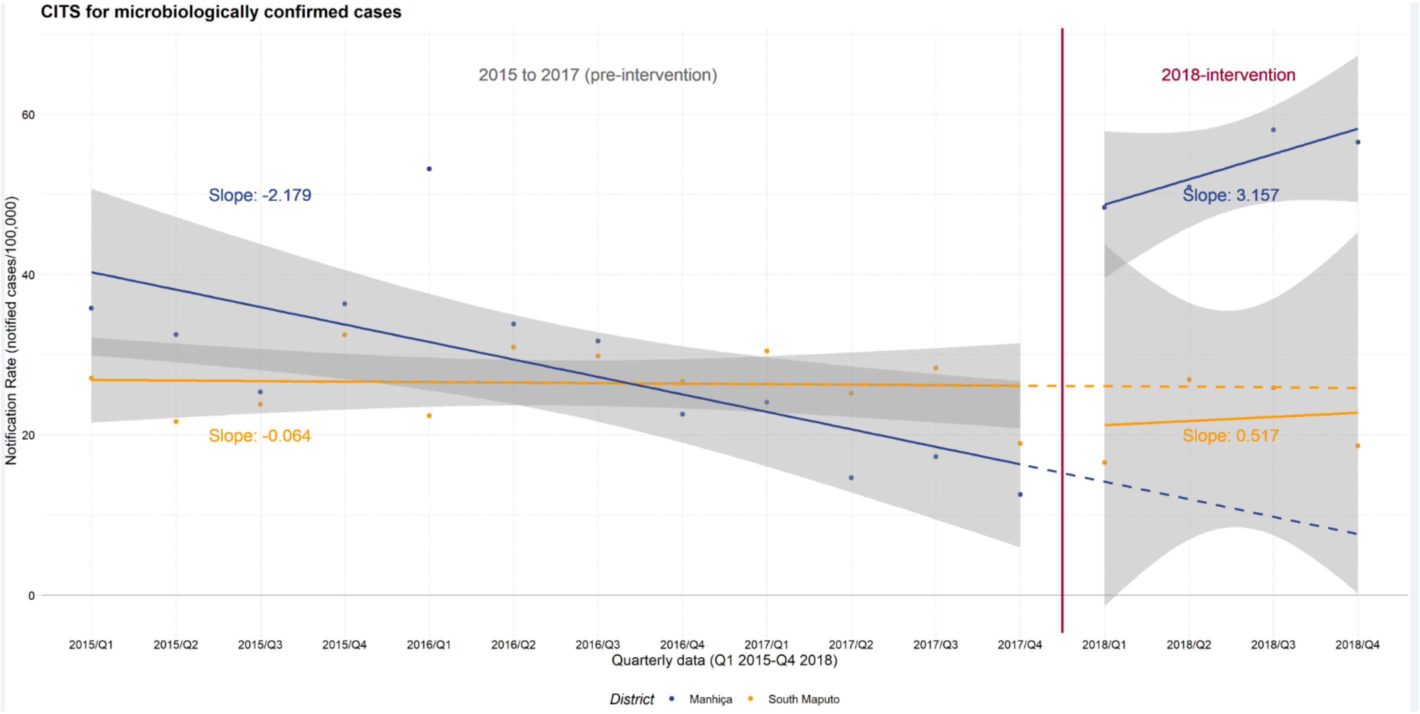

Furthermore, some minor comments require attention before the acceptance of the manuscript.

Reviewers' comments:

Reviewer #1: The authors addressed the majority of my comments from the earlier review. My major concern is that the concluding statement in the abstract is not supported by the data presented. The abstract states "The CITS model showed an increase of laboratory confirmed TB cases in the intervention district, compared to the control area". However, Table 4 shows no statistically significant difference in step change or intervention trends between the two areas. Please rephrase this statement so that it is aligned with the results.

Response: Thank you for bringing up this to our attention. In response to this and the Editor´s similar comment, we have carefully considered the feedback and have made the due adjustment in the abstract. Now it reads: *During the intervention period, a reversal of the previous negative trend in lab-confirmed case notifications was observed in the Manhiça district. However, the CITS model did not show any statistically significant difference when compared to the control area*

Minor comments include:
- Of the 10 asymptomatic PLHIV diagnosed with TB, 7 were bacteriologically confirmed (Line 230). Please explain the justification for TB diagnosis of the 3 asymptomatic PLHIV with no bacteriologic confirmation.

Response: Thank you for this contribution. We have added information about the clinical diagnosis in PLHIV in the fourth paragraph of the methods sections. Now it reads (line 160/161 track-changes version): "*Clinical diagnosis was established by clinicians as per national guidelines (symptoms screening and X-ray interpretation).*" In addition, we have referred to this section in line 234.

- **In line 258, please specify that 1010 refers to index cases and 7895 refers to people who were identified as contacts.**

Response: Thank you for this suggestion. We have clarified this sentence. Now it reads: "*From those 1010 ICs, 7895 identified contacts were screened (…)* "

- Please note that the use of p-values in Table 2 is not consistent with best statistical practice (see Wasserstein et al. below). I recommend removing the p-values from Table 2. If the authors insist on using p-values, they should be consistently reported for all variables. At present, p-values are presented for some variables but not all in Table 2 (e.g. Prevalence of HIV, age group).

- 1.Wasserstein RL, Lazar NA. The ASA's Statement on p-Values: Context, Process, and Purpose. The American Statistician. 2016 Apr 2;70(2):129–33.

Response: We appreciate the reviewer for sharing their point of view, and we understand the concern. We agree that relying solely on p-values from univariate analysis is not sufficient to draw strong conclusions. However, it is a common practice to include these values as an initial indication of the potential association of variables in relation to the outcome. Taking the reviewer's feedback into consideration, we have added the missing p-values that were not included in the previous version. We have removed the information of median age difference among women and men to simplify the table.

We would like to point out that we did not apply a statistical test for X-ray results due to the large amount of missing data in this variable.

- Line 414 states that the NNS shows higher effectiveness of HC screening. However, NNS is a measure of efficiency, not effectiveness. I recommend changing the term "effectiveness" to "efficiency" or "yield".

Response: We appreciate your recommendation and we have substituted the word '*effectiveness'* by '*yield'* in the new version.

- Line 434. I suggest changing "NNS to find a man/woman" to "NNS to find a TB case among men/women".

Response: We thank the reviewer for this comment. NNS women/men indeed means NNS to find a woman/man with TB among all screened. As a result of a previous comment, we removed NNS for women among women and men among men in the previous version.

In order to make it more understandable now, we have modified this sentence and now it reads: "*NNS to find a man/woman among screened: 430 vs 226"* (Line 440-441 track-changes version)

- It would be helpful to know if the sex differences in NNS among community contacts were observed in prior TB ACF studies. Suggest adding context to this finding based on existing literature, if any.

Response: We appreciate this important comment. Indeed, we conducted an extensive search on sex stratification and resulting NNS in TB-ACF studies, and we did not find available data.

Nevertheless, we fully acknowledge the importance of sex stratification in the statistical analysis. The interpretation of it is crucial to ensure a comprehensive understanding of the underlying determinants of health, considering gender differences. Recognizing this, a subsequent secondary analysis on gender related factors is on its way. We will explore if there are any relevant data or insights regarding sex stratification and its impact on NNS in ACF studies for TB, aiming to shed light on potential gender differences and their implications.

- It seems that the uploaded csv file contains dates of birth, which could be used to identify study participants. Suggest removing this column from the shared data file.

Response: Thank you for arising this important point. We have now removed this column and updated the dataset.

Once again, we appreciate the reviewers' time and effort in reviewing our work.
We wish to thank for the opportunity to respond and for considering this article for publication in PLOS Global Public Health.

Sincerely,

Dr. Alberto L. García-Basteiro, on behalf of the authors
