# Supplementary material for: Improving tuberculosis case detection through contact risk stratification by Xpert MTB/RIF Ultra and spatial parameters: Evaluation of an innovative active case finding strategy in Mozambique (Xpatial-TB)
Source: PLOS Glob Public Health. 2024 Feb 9;4(2):e0002789. doi: 10.1371/journal.pgph.0002789 (PMC10857722; doi:10.1371/journal.pgph.0002789)
Supplement: Attachment — Submitted filename: Response_to_reviewers_May2023_PGPHD-22-01781.docx [file pgph.0002789.s007.docx]

Manhiça, 3 April , 2023

Dear Dr Raquel Muñiz-Salazar

Thank you for the opportunity to revise and resubmit the manuscript entitled ***“Improving tuberculosis case detection through contact risk stratification by Xpert MTB/RIF Ultra and spatial parameters: Evaluation of an innovative active case finding strategy in Mozambique (Xpatial-TB)”*** which we re-submit for publication in *PLOS Global Public Health* after receiving feedback from the reviewers and editorial team. We have carefully reviewed the various comments issued, and effected changes to the manuscript where appropriate. Below, please find our itemized responses.

To expedite the review of this revised manuscript, we have uploaded two versions of the manuscript. Version 1 ‘Manuscript’ (‘clean version) that includes the unmarked version of the revised manuscript, and version 2 ‘Revised Manuscript with Track Changes’, as requested, to visualize where changes have been made without difficulties.

**Editor´s comments:**

In response of the issues related to Journal requirement:

1) The Financial Disclosure statement and the Competing Interest statement have been modified and updated accordingly. Thank you for your consideration.
2) Concerning data availability, we have attached a csv.file including the study dataset, a brief dictionary, and the National TB programme registries to replicate the ITS/CITS analysis.
3) Thank you for noticing the absence of the list of legends. As requested, we have included a list of legends at the end of the manuscript, including tables, figures and supplementary material.

**Additional Editor Comments:**
**The manuscript is attractive and well-designed but lacks an analytical approach. This relevant study combines Xpert Ultra semi-quantitative and spatial parameters on tuberculosis case notifications in a high TB-HIV burden district of Southern Mozambique.**

Response: Thank you for the overall positive reaction to our manuscript. We hope that this new version will shed light on the analytical approach and facilitate the understanding of the methods applied and results.

**Reviewers'** **comments**:

**Reviewer #1: Overall, the authors submitted a well-written manuscript describing the programmatic outcomes of a novel active case finding method for TB. Major comments and methodological concerns include:**

**- Conclusion in abstract states that the intervention led to increase in TB case notifications. This seems like an overly strong statement of causality given that the differences between control areas could not be established. Therefore, the increase in TB case notifications could be due to secular trends and possible detection of cases that would have also been detected via passive case detection. While I believe ACF likely had a beneficial effect, I think the conclusion should be more nuanced.**

Response: Thank you for your overall positive reaction about the standing of the paper, and for bringing up this important concern in regards to the conclusions. Following your suggestion, we have deleted the closing sentence and focused on proven benefits of the strategy. Now it reads: *Paediatric population benefited the most from the ACF strategy and HCs screening seemed an effective intervention to find microbiological confirmed cases in early stages of the disease.*

**- I think the manuscript could be strengthened by greater attention to the possible impact of over-diagnosis of TB in the study. It is striking that <40% of index cases were microbiologically confirmed TB. The relatively low yield of TB among contacts could also reflect that a significant portion of the index cases did not have TB. On the other hand, nearly half of contacts diagnosed with TB were not bacteriologically confirmed, which could have led to over-estimation of the yield of ACF.**

Response: Thank you for your comment. Indeed, we also agree with the low proportion of confirmed cases among index patients. However, we would like to point out that the study did not intervene in the diagnosis flow of index cases, which followed the procedures of the national TB programme. As stated in the discussion, this striking data is aligned with reported figures. In 2018 (year of the study) Mozambique confirmed just 39% of TB cases (Global TB report 2019).

We understand your comment although we theorize differently. Instead of over-diagnosis we suspect an important proportion of hidden patients because of the following: 1) Mozambique is a high TB and HIV burden country; 2) a widely-applied screening found more lab-confirmed cases; 3) other neighbouring countries have achieved better figures for microbiological confirmation. In the end, in high-burden settings, everyone is at risk of being infected almost everywhere.

**A few suggestions:**

**o In Methods, please describe additional details about the TB diagnosis process in Mozambique with a focus on three districts. For example, if Xpert MTB/RIF was not being used in the 2 control districts, were sputum microscopy primarily used? What are the clinical diagnostic criteria for laboratory unconfirmed TB? Likewise, for Manhica, Xpert Xpert/MTB Ultra was used as the “front line” test. What was the process of diagnosis among Xpert negative cases?**

Response: Thank you for arising these important points. In regards to control districts, we have rephrased the second paragraph of Methods (line 133-136). Now it reads: *We chose notification data of 2 similar rural districts in Maputo province (Namaacha and Matutuine. that do not limit with our district* *Those areas applied smear microscopy as laboratory test for TB confirmation and did not participate in other ACF projects in place or implemented the use of Xpert Ultra during 2018.*

In addition, more details about study procedures can be updated in Supplementary material (S1). Now it reads: *Patients were managed according to the National Tuberculosis Programme (NTP) guideline and they started on TB treatment shortly after TB confirmation or high diagnostic suspicion (clinically diagnosed cases).*

*.***o Please consider performing sensitivity analysis limiting the data to lab confirmed TB. Do the findings and overall conclusion differ?**

Response: Thank you for your suggestion. Indeed, we already assessed the diagnosis performance of the Xpert Ultra compared to Xpert MTB/RIF and culture as reference standard. Results are published in the European Respiratory Journal *(Saavedra B, Mambuque E, Nguenha D, et al. Performance of Xpert MTB/RIF Ultra for tuberculosis diagnosis in the context of passive and active case finding. Eur Respir J. 2021;58(6):2100257. Published 2021 Dec 23. doi:10.1183/13993003.00257-2021).* This analysis added evidence on the improved sensitivity of Ultra and support its use in different case finding scenarios

**o Please add a description of the effect of possible inclusion of non-TB index cases in the limitations.**

Response: We thank you again for this suggestion. As stated before, we hypothesized that in a setting as Manhiça is, the risk of having TB is as high as we would not consider the proportion of likely over-diagnosis high. Nevertheless, we have added a sentence in the discussion to point out this possibility. Now it reads: *It can be argued that the small window screening period used (3months), might be too short to find recent contacts (35), or the existence of incident cases misdiagnosed as clinical cases*

**- Given the objective of the study and data collected, I question whether ITS is the appropriate analytical method for this study. It seems that a simple comparison of the notification rate before vs. after and between intervention and control districts would be sufficient. I find the ITS findings overly difficult to interpret. I suggest removing the ITS analysis from the manuscript. However, it the authors do keep ITS analysis, please consider the following:**

**o Please explain your hypothesis of your ITS analysis. Would you expect a step change or slope increase? Why?**

Response: Thank you for your interesting comment and we apologise if we were not able to reflex the rational of the analysis.

We chose this specific analysis based on the literature about ITS/CITS (ref 25 *The use of controls in interrupted time series studies of public health interventions,* among others). Briefly, we considered the ideal method to evaluate results because of the following:

1) This design is generally applied to interventions introduced at a known point in time.
2) We collected data at regular intervals over time
3) This is not only a way to compare the intervention before & after, but also a strategy to avoid misleading conclusions due to random or secular trends.

We hypothesized that notification rates would increase because of both, the use of a more sensitive diagnostic test, and the ACF activity itself.

In order to clarify the rational of this analysis we have included this explanation in S2 Supplementary material 2. S2. Interrupted time-series for single and multiple-group comparison. Besides, we have rephrased lines 171-172. Now it reads: *iii) conducting an interrupted time series analysis with and without control (CITS/ITS) of aggregated quarterly TB notification cases(25). Likewise, we aimed to compare data before and after the intervention, hypothesizing that potential increase in notification rates would not be related to random temporal trends...*

**o Tables 3 and 4 include NR Ultra and NR Xpatial coefficient estimates. It is unclear from the methods how you can distinguish the effect of Ultra vs. the combined intervention since both Ultra and ACF were implemented at the same time. Please clarify in the Methods and on the table.**

Response: Thank you for noticing that this important information was missing. Briefly, for the first ITS analysis we did not count cases derived from the ACF strategy and we just made use of cases notified by the NTP (index cases). As detailed in methods and Supplementary material, during 2018 the Xpert MTB/RIF was replaced by the Xpert Ultra throughout the district, thus we were able to easily distinguish the two strategies. We then performed a second ITS analysis by merging the data with cases arising from the ACF project.

Regarding the CITS models, given that results are similar and in order to simplify table 4, we have just shown CITS parameters for the complete intervention. We have clarified those steps in methods and now it reads: *We evaluated both, ITS-1: the introduction of the Xpert Ultra as the initial diagnostic test in the entire district (based on cases reported by the NTP), and ITS-2: the complete intervention (the use of Xpert-Ultra and ACF activities) by merging previous information with cases derived from ACF activities.* *In addition, a control area was chosen as a second counterfactual to perform a CITS analysis* (lines 181-187)

Tables 3 and 4 have been also modified accordingly. Titles, notes and figures have been reviewed.

**o Figure 4 is very difficult to understand. Please add details to the figure legends and add labels to the figure as needed to clarify what is being presented. Also, please confirm that the colours are correctly labelled. As it stands, it appears that there was a big step increase in case notification among control districts post-intervention, but a decrease in Manhica.**

Response: Thank you for realising the error. Indeed, colours were not correctly labelled. In addition, and also aligned with reviewer 1´s comments, we have maintained just the CITS analysis for the complete intervention (use of Xpert Ultra+ACF activities). As stated previously, this has been updated and clarified in methods and Figure 4 have been simplified. We have also included some more details in footnotes.

**Minor comments and feedback include:**

**- Line 72 The citation field needs to be updated.**

Response: Thank you for noticing. We updated the reference.

**- Line 93 “quality or” should be corrected to “quality of”**

Response. Thank you for the correction. Now it reads: “there are some concerns on the quality of TB diagnostic and reporting services”

**- Line 105. I believe the authors meant “exposure” not “exposition”**

Response. Thank you for your suggestion. We have changed it accordingly

**- Line 118. The study is described as a “prospective controlled intervention study”. It is unclear from this definition, how the study is controlled. I suggest adding details about the methods of control here or remove “controlled” from this description.**

Response. Thank you for your suggestion. In order to clarify the study design, we have slightly modified this paragraph. Now it reads: *Over a period of 12 months (January-December 2018) the whole district of Manhiça participated in the Xpatial-TB project (TB REACH, wave 5, funded by the Stop TB partnership). This was an intervention study which introduced 1) an ACF initiative, 2) the novel Xpert Ultra assay as the frontline test for active TB diagnosis (as it has been described elsewhere (23)).*

**- Figures 1 and 2 are very clear and helpful for understanding the study. Thank you for including them.**

Response. Thank you for your positive comment.

**- Line 143 “Once and IC” should be “Once an IC”**

Response: Indeed, this sentence had a mistake. The text has been updated.

**- Line 143 and throughout paper: Since HDSS data were used to identify neighbors for screening, I believe it’s incorrect to call them “contacts”. In general, “contacts” are used to refer to people who had physical contact with the index case.**

Response: Thank you very much for your comment. We would like to explain that this is the terminology used in the proposal and project activities. Actually, we called them ‘community close contacts’ based on the hypothesis that neighbours would have some type of physical contact. In rural communities, such as our, people share spaces in compounds to develop daily activities such as plays, food time and so on. Likewise, our target population (TP) was all household contacts (HC) and community-close (CCC). Those CCC were assumed to be located near the index cases, and were depicted in spatial analysis conducted at the site.

Nevertheless, we agree that this is not clear in the text. We have rephrased line XX in methods an updated this term throughout the text. Now it reads: *ACF activities*: Once an IC was identified, a list of households (HCs) and close community contacts (CCs, eligible neighbouring residents) was generated based on HDSS registers (line 137-138)

**- Please justify why epidemiological contact history was not taken (or if it was, why it was not used to guide contact investigation).**

Response: Thank you for your comment. Indeed, this is the rational of the entire intervention. We presume that all those who were screened would have had contact with the index at some point. Relying on that, we followed the study algorithm (Fig 2) to investigate TB.

**- Line 178. Please describe the specific variables of the TB care cascade and process indicators that were included in the outcome analysis.**

Response: Thank you for this comment. We have included a sentence in lines 166-168, describing the specific variables of the TB care cascade and process indicators used in our analysis: number of participants screened; number of participants enrolled, number of participants who provided sample, and number of patients diagnosed.

**- Line 209. Continuum should be continuous.**

Response. Thank you for your suggestion. Now it reads: “Continuous variables were compared through means t-test and categorical variables through chi-square test.”(line 209).

**- The use of existing community census data to guide ACF is novel. Please add details on the outcome of that effort. For example, what was the average number of HH contacts and neighborhood residents screened per index?**

Response: Thank you for your questions. Indeed, this information may be inferred from Fig 1. Although we found approximately 4 HCs (4394/1010) and 6 CCs (6373/1010) eligible for screening per index case, we finally screened 3.1 HCs and 4.7 CCs in average. We have included this detail in line 220-22.

**- Table 1. If NNS women (8) and NNS men (9) represent the total men and women needed to screen to diagnose one women or men, they do not seem meaningful to me. Please justify why this was calculated or remove.**

Response: We thank you for this comment. Indeed, we have noticed some differences in the diagnosis of TB based on sex. Nevertheless, we agree that this point would need a detailed explanation, thus we have removed this part from here.

**- In Results, Please consider placing Characteristics of cohort before the description of contact screening results.**

Response: We thank you for this input. Nevertheless, we have kept the order as we deem the first section of utmost importance to understand which cohorts we are describing later.

**- Results. Please show the yield by Xpert Ultra bacillary burden categories.**

Response: We thank you for this suggestion. Indeed, this data can be found in table 2, stratified by PCF or ACF cases and also separated by HCs or CCs. We did not detail this information within the text in order to save space. Despite that, we specified that in lines 274-275: *More than half of cases was bacteriologically confirmed (52.8%, 47/89), and 74.5% (35/47) of them relied on Ultra´s lower categories (very low or trace).*

**- Results. Please show yield by type of sample (e.g. sputum, urine, stool)**

Response: We thank you for this comment but 99% of samples were respiratory and just 3 cases were confirmed by stool in paediatric population. This is the reason why we did not specify this within the manuscript

**- In Discussion, please place NNS in context with prior household contact studies.**

Response: We have rephrased line 414 and added a reference: ‘*Based on NNSs (common indicator to evaluate ACF activities (31)), the effectiveness of HCs screenings was considerably higher than for CCs.’*

**- Limitations. Please include the lack of epidemiological contact information available to guide ACF.**

Response: As explained in a previous comment, we did include the epidemiological contact information to guide ACF, on the premise that we considered all those investigated to be index contacts.

**Reviewer #2: Comments to Authors:**

**Introduction**

**1. Line 67, cite latest TB prevalence rates from latest WHO report**

Response: Thank you for noticing. We updated the reference and data to the latest Global TB report.

**2. Line 90, Use full form of any terminology at its first use.**

Response. Thank you for realising. We have developed the acronym of “TB-MDR” and “TB/HIV” in the text.

**3. Line 91, what do you mean by detection gap?**

Response: Thank you for your question. The WHO estimates that 48% of TB cases were not diagnosed from TB, which is called ‘detection gap’.

**Methods**

**4. Xpatial Strategy: Fig.1: provide the footnote.What does numbers 1,2 and 3 in superscript forms within the fig 1 indicate?**

Response: Thank you for your question. Indeed, this information was detailed but not as a footnote (this was in the title). We have changed it accordingly

**While dividing the Xpert Ultra results in two categories, what does low category in both bifurcations means? Explain**

Response: Thank you for your noticing this error. The low category should be included just on the left path. We have corrected it in the new figure.

**What do you mean by clinical TB? The resolution of figure is very low.**

Response: According to international guidelines, those cases who are not microbiologically confirmed but who are diagnosed under clinical criteria may be call ‘clinical cases’. The information in regards to specific diagnostic procedures is developed in methods and Supplementary material (s1)

**Results**

**5. Table 1: What was the purpose behind screening gender-wise NNS (NNS women, NNS men)? Why NNS women among women and men were selected top screen? The purpose of superscript number is not mentioned in footnote.**

Response: Thank you for your comment. Indeed, we believe that the absence of sex-disaggregated data has direct consequences, biasing results and conclusions. Given sex-differences in the number of TB cases worldwide, we consider that basic figures should be shown stratified by sex. We know that TB prevalence and incidence is different for men and women. Nevertheless, we understand that this specific point would need a specific discussion and this is why we have removed NNSs for women among women and men among men

**6. Sociodemographic characteristics of index cases: Line 264-267, “In 2018 1020 incident TB cases ……… women and men”, statement is not clear.**

Response: Thank you for this suggestion. We have already removed this sentence in order to avoid confusion

**7. Line 267-269, “PLHIV …… pulmonary disease”, make the statement clearer.**

Response: We have rephrased this initial part to make it clearer. Now it reads: *In 2018, 1020 incident TB cases were reported by the NTP. Individual information of those identified passively was available in 1010 patients (signed informed consent); the median age was 39.5 [IQR 30.1-52.4], 53.7% (543/1010) were male, and PLHIV accounted for 63.5% of the total of ICs (641/1010). Pulmonary disease was present in 92.7% (936/1010) of them.*

**8. Numbers are not matching in table 2 and text for microbiological confirmation and confirmed HIV negative and positive and so on.**

Response: Thank you for arising this error. We have changed number accordingly.

**9. Line 290, Pediatric cases: children <15 or <12? Explain.**

Response: Thank you for your question. As specified within the text and table, we considered pediatric population those participants under 12 years old. However, when we categorised the variable ‘age’, we used the upper limit of 15 in order to make proportional intervals.

**10. Table 3 make separate columns for pre and post intervention for NR ultra and NR Xpatial**

**11. Line 346-349, p values and β1 and β3 values seems confusing. Please make a clear table 3**

**12. Line 367, number are not matching with the table 4. Make table 4 clear for different districts and pre post intervention periods.**

Response: Thank you for your suggestions. Considering your comments 10-12, and the other reviewer´s suggestions, we have modified tables 3 and 4 to make them easier to understand.

**13. Figure 4 make resolution clearer and explain the graph or slope how it is showing a positive trend.**

Response: Thank you for comment. We have improved Fig 4 and added information in footnotes to explain the plot.

**14. There is no molecular evidence whether these HCc and CCs belong to the true index cases they were tracing, or contacts got infection from some other sources if they had migrated or travelled during this time.**

Response: Thank you for this important comment. Indeed, we have now the genomic information of this study and we are preparing the manuscript to show the molecular epidemiology of intervention data. This is the reason why we arise this point in the discussion and we state as follows: *we wondered whether TB cases identified through CCs were actually cases epidemiologically linked to the IC (or, alternatively, co-prevalent cases)*

Discussion

**15. Your objective is to see the trend in improvement of TB case detection using ACF strategy not to see the index cases trend in pre- and intervention period. What’s the purpose of looking at this trend in pre-intervention period?**

Response: Thank you for your interesting comment and we apologise if we were not able to reflex the rational of the analysis.

We chose this specific analysis based on the literature about ITS/CITS (ref 25 *The use of controls in interrupted time series studies of public health interventions,* among others). Briefly, we considered the ideal method to evaluate results because of the following:

1) This design is generally applied to interventions introduced at a known point in time.
2) We collected data at regular intervals over time
3) This is not only a way to compare the intervention before & after, but also a strategy to avoid misleading conclusions due to random or secular trends.

We hypothesized that notification rates would increase because of both, the use of a more sensitive diagnostic test, and the ACF activity itself.

In order to clarify the rational of this analysis we have included this explanation in S2 Supplementary material 2. S2. Interrupted time-series for single and multiple-group comparison. Besides, we have rephrased lines 171-172. Now it reads: *iii) conducting an interrupted time series analysis with and without control (CITS/ITS) of aggregated quarterly TB notification cases(25). Likewise, we aimed to compare data before and after the intervention, hypothesizing that potential increase in notification rates would not be related to random temporal trends...*

**16. “Indeed, 13.7% of notified TB cases reported on Ultra's new lowest category, "trace". If the former Xpert assay had been used instead, it is likely that those cases would have not been confirmed (and a proportion of them would have been not diagnosed for TB). The initial debate on the significance of trace results for the Xpert MTB/RIF Ultra cartridge seems to have achieved a certain consensus. The latest recommendations advocate against retesting in favor of a cautious assessment of the clinical presentation and individuals’ characteristics “. Of course, new technology will have some improvement tin case detection rather than old. What’s the reason behind bringing this discussion here?**

Response: Thank you for arising this debate. Indeed, all diagnostic tests need to be validated under field conditions. Initial studies to evaluate the diagnostic performance of the Xpert Ultra were contradictories about the interpretation of trace results. The scientific community has debated extensively on this issue and at the time of the study the interpretations was not clear yet. This is the reason why we brought this point here.

In addition, we have reviewed the wording and grammar of the text, applying minor changes throughout the document, and we have included the acknowledgments section before references

We wish to thank for the opportunity to review this article for publication in PLOS Global Public Health.

Sincerely,

Dr. Alberto L. García-Basteiro, on behalf of the authors
