# Supplementary material for: Improving tuberculosis case detection through contact risk stratification by Xpert MTB/RIF Ultra and spatial parameters: Evaluation of an innovative active case finding strategy in Mozambique (Xpatial-TB)
Source: PLOS Glob Public Health. 2024 Feb 9;4(2):e0002789. doi: 10.1371/journal.pgph.0002789 (PMC10857722; doi:10.1371/journal.pgph.0002789)
Supplement: Attachment — Submitted filename: Response_to_reviewers_December2023_PGPHD_final_ALGB.docx [file pgph.0002789.s009.docx]

Manhiça, 8 December, 2023

Dear Dr Cesar Ugarte-Gil,

Thank you for the opportunity to revise and resubmit the manuscript entitled ***“Improving tuberculosis case detection through contact risk stratification by Xpert MTB/RIF Ultra and spatial parameters: Evaluation of an innovative active case finding strategy in Mozambique (Xpatial-TB)”*** which we re-submit for publication in *PLOS Global Public Health* after receiving the feedback from the reviewers and editorial team. We have carefully reviewed the new comments and effected changes to the manuscript where appropriate. Below, please find our itemized responses.

To expedite the review of this revised manuscript, we have uploaded two new versions of the documents;

i) *‘Manuscript_December2023’* (‘clean version) that includes the unmarked version of the revised manuscript;
ii) *‘Revised_Manuscript_Track_Changes_December2023’ with all marked changes.*
iii) *‘Supplementary_material_track_changes_December2023 (clean and track-cahnges version*

to visualize where changes have been made without difficulties.

**Additional Editor Comments**

**First, apologies for the delayed response to the authors. The majority of the comments and concerns were solved by the authors, but a reviewer provides some fair comments that I consider important to address and/or explain as potential limitations before to accept it for publication. Considering the delay in the process, as a Editor I will check them as soon the authors submit them**

Thank you for your feedback on our manuscript. We appreciate the time and effort you dedicated to reviewing our work. We are confident that upon reviewing the updated version, you will find the manuscript suitable for publication.

**Comments to the Author**

**Reviewer #3: Synopsis**

**Major issues:
1. TB case definition and diagnosis: Only 375/1010 (37.1%) of the index cases (ICs) had laboratory-confirmed TB. Furthermore 568 (89%) of the 635 clinically diagnosed ICs had actually a negative Xpert Ultra result (from data file provided, numbers not included in the manuscript). 49/357 (13.7%) of the laboratory-confirmed IC cases were classified as laboratory-confirmed TB on the basis of trace positive Xpert results without taking previous TB history into account. As a result of the above, it is very uncertain whether the IC, or the ACF, actually had TB, particularly given the very high proportion of ICs which had a negative Xpert test.**

Response: We do agree with the astonishing low confirmation rate. Nevertheless, this is a well-known and extensively debated issue for Mozambique. In 2018 (intervention year), the proportion of reported cases with bacteriological confirmation was 39% in the country, followed by 37%, 35% and 38% in 2019, 2020 and 2021 respectively. The actual TB report revealed better data for 2022 (43%), but still far from the global 63%.

We have decided to highlight this issue and we now mention it in the introduction (lines 85-88). The routine diagnostic strategies used by National TB programmes, based on passive-case finding, miss many cases, especially in high-burden settings. For this reason, ACF strategies are key to contribute to national control programmes. Indeed, the *Consolidated Guideline on tuberculosis. Module 2: screening,* recommends that ‘household contacts and other close contacts of individuals with TB disease, should be systematically screened’, and also that ‘systematic screening for TB disease may be conducted among people with a risk factor for TB in countries where the prevalence of TB is high, irrespective of bacteriological confirmation.

We thus designed an algorithm in which the screening of close contacts was just planned in those cases in which the index case was bacteriologically confirmed and high a higher bacteriological burden as observed in Xpert Ultra results (excluding those with an Ultra result *trace* or *very low*). Furthermore, all household contacts were screened as it is currently recommended in many national programmes, regardless the status of the index case.

The low confirmation rate has been stalled during years in Mozambique, and one of the objectives of this project was to look for innovative ways to improve the microbiological diagnosis of TB. Interestingly, 47.2% of new cases identified by the Xpatial-TB project derived from non lab-confirmed TB cases, supporting the strategy of household screening, irrespective of microbiological result.

Finally, we also debated about the management of patients with a positive but ‘trace’ molecular test. In the discussion section, it is now stated that : ‘*The initial debate on the significance of trace results for the Xpert Ultra cartridge seems to have achieved a certain consensus. The latest recommendations advocate against retesting but in favour of a cautious assessment of the clinical presentation and individual characteristics’.* In our case, all individuals classified as a TB case underwent meticulous examination, including clinical assessment, X-ray, and investigation into the history of a recent TB. Those with a documented previous (and recent) episode of TB were excluded from the analysis. Consequently, we have confidence that those referred cases indeed represent recent and real TB patients.

**2. Analytical methods: The authors note a definitively non-linear trend in notification rates (NR) in pre-intervention period (2015-2017) within the intervention district (lines 295-297) with NR increasing between 2015 and 2016 but declining in 2017. The use of linear regression ITS/CITS is therefore inappropriate as it averages the pre-intervention trend across the period and is likely the main driver of the large level decrease (B2 coefficients, table 3) in the in ITS-1 and ITS-2 models. While non-linear methods may somewhat address the issue, there is another underlying problem in that the data for the 2015 quarters were derived from aggregated reports from the NTP whereas the 2016-2017 (and ?2018 data) were obtained from individual level data raising potential concerns about different data processing and therefore comparability of the newer data with the 2015 data.**

Response: Thank you for bringing this to our attention. We have performed an extensive investigation before applying the ITS and CITS analysis. The ITS design is a quasi-experimental approach to evaluate health interventions introduced at a population level over a defined point in time. For that, one of the most common models applied in the literature is the segmented linear regression. Although this term ‘linear regression’ might suggest a linear pattern, it refers to the use of line segments to model the trend before and after the intervention. Some other studies are published following this methodology, although the data points do not show a clear linear pattern. Nevertheless, we are aware that having just four data points in the intervention limits the precision and power of the analysis and difficulties the estimation of the parameters. This is well acknowledged in the discussion.

The alternative CITS minimizes this limitation with the introduction of a control area as a second counterfactual to introduce a pooled control series. Additionally, this inclusion might minimize other confounder such as external events that might have affected notification rates during the intervention period.

Despite of this consideration, the CITS model did not solve the lack of statistical significance, and we discuss those limitations in the fourth paragraph of the discussion (lines 395-404, track-changes version)

Lastly, in order to provide a better explain the rational of the analysis, we have made some editions in the methodology of the manuscript (Section *Data analysis*). Basically, we have divided the explanation of the different models and added the information explained above (lines 171-214 track-changes version)

Concerning the data used for the analysis, we apologise for the confusion. We do agree that the explanation about the source of the data was not clear in the Supplementary material. We have moved this information to a new paragraph within the S2. ITS and CITS model, and we have clarified it. Now it reads:

*S2.3. Databases: Since 2016, the NTP counts with digital registers of TB notifications (SIS-MA) as aggregated data reports that are subjected to data cleaning and duplicate removal. This information derives from individual TB case reporting obtained from the data collected on paper-based registries (official NTP books). Therefore, the number of cases for 2015, 2016, 2017 and 2018 was obtained from quarterly aggregated information provided by the national TB surveillance system (official reports).*

**3. Selection of control areas: The study combined data from 2 non-invention areas to form a control for the CITS analyses. It is unclear how or why these areas were selected and more information is required. (As an aside it appears from the supplementary material that a different control area “Magude” which is in a very different area may have originally been considered.) The substantial differences in the pre-intervention intercepts and pre-intervention slopes (B4 and B5 coefficients respectively, table 4) raise concerns about the conclusions drawn from the CITS models. Alternative control areas which result in B4 and B5 coefficients with higher p-values should be considered if at all possible.**

Response: Thank you for this contribution .We apologise for the error present in the previous version of the Supplementary material, that we have already corrected (Supplementary material 2). Additionally, we have introduced a new section in S1, providing comprehensive information about the control area, and elaborating on the selection process. Briefly, our criteria to choose control districts involved the identification of rural areas (similar to Manhiça), which did not use Xpert Ultra as a frontline test for TB diagnosis in 2018. Moreover, we ensured that those areas did not share borders with Manhiça, mitigating the risk of case transfer (a spill over effect from neighbouring district). Particularly, this last consideration led to the rejection of the initially considered area, Magude. We personally verified the diagnostic flow of Namaacha and Matutuine with the NTP, and population data was sourced from the national institute of statistics.

Upon further review, we have decided to revised the text of the S1 section to increase clarity. New edits include the refining study procedures section (PCF activities, sample collection and laboratory procedures), and the incorporation of a map illustrating health units and hospitals within the districts. The track-changes version is uploaded for transparency on the text that has been modified.

We also agree with your comment on ‘the substantial differences in the pre-intervention intercepts and pre-intervention slopes (…)’. Unfortunately, after careful assessment we could not find other districts with similar geographic and demographic characteristics without molecular testing during the year of the study in the province. As discussed earlier, we are cognisant of the limitations inherent in our model that, to a large extend, are probably due to the absence of additional data points after the intervention. Instead of considering the selection of the control area as critical factor influencing the model, we are inclined to believe that the issue leading to non-significance in the coefficients might be attributed to the confidence intervals calculated for each regression model.

**Minor issues:**

**1. Include map of the intervention and control areas.**

Response: Thank you for your suggestion. Following the recommendation, we have included two maps in the Supplementary material (S1). The first map focus on the study area, while the second map highlights the control area for reference.

**2. The paper requires substantial editing for grammatical and language issues. A few examples are given below (by no means an exhaustive list):**

**a. Abstract line 39: Change “rolling-up” to “rolling out”
b. Abstract line 43: rephrase “on Xpert Ultra ´s result of the IC “with “on the Xpert Ultra result of the IC”
c. Abstract line 43/44: rephrase “population density of the area where the IC lived in” to “population density of the area in which the IC lived”
d. Abstract line 45: Edit “asked for providing” to “asked to provide”
e. Line 110: “approximately 204,953” – suggest round to nearest 1000 to avoid spurious accuracy.**

**f. Line 260: “being the age distribution different between HCs and CCs” is unclear**

**g. Line 287: Write “BK” in full as abbreviation not used elsewhere**

Response: We thank the reviewer for the initial suggestion and the corrections provided. All noted issues have been addressed accordingly. Additionally, we conducted an exhaustive review throughout the entire document to identify and rectify any remaining grammatical issues identify and correct any grammatical issue.  **3. The proportion of ACF requiring sputum induction with portable nebuliser would assist in interpreting the feasibility of the ACF intervention in settings without portable nebulisers.**

Response: Thank you for this interesting comment. Unfortunately, this specific information is not available. Based on our study experience, sputum induction was predominantly employed in cases without respiratory symptoms. The production of spontaneous respiratory secretions was challenging in the absence of cough.

**4. Line 194 in methods: ”Autocorrelation was tested by Cumby–Huizinga” but results of this test are not presented.**

Response: Thank you for noticing. After an in-depth review of the methodology applied, we have considered the use of the Breusch-Godfrey test, as is more commonly employed to test autocorrelation in the residuals of a segmented linear regression model. Utilizing the package ‘lmtest’ and the function ‘bgtest’, we have now added p-values for the residuals of each model adjacent to the respective tables.

**5. Line 254: “Just 1.1% (11/1010) showed resistance 254 to rifampicin by Ultra testing” but denominator includes both those with negative Xperts and without Xpert tests.**

Response: Thank you for bringing our attention on this. We do agree that it would be more accurate to calculate the proportion of rifampicin resistant using just Ultra positive cases in the denominator. We have now updated the percentage. Now it reads : Just 3.1% (11/357) showed resistance to rifampicin by Ultra testing

**6. Table S1: Screening radius for medium density areas not provided.**

Response: Thank you for noticing this error. It has been corrected accordingly.

**7. Supplement S2.1 and S2.2: it appears that the coding for the intervention period dummy variable Xt: could be incorrect (dummy variable; pre-intervention (before 2018)=1/intervention(2018)=0)” as the B coefficients will be the value in the pre-intervention period where X==1 and 0 in the post intervention period where X==0.**

Response: Thank you again for your detailed review. The coding was correctly described in the manuscript; however, we acknowledge this error in the supplementary material. It has been rectified and the information is now correctly written.

**8. Reference issues (again not a complete list)**

**a. Line 114: reference 21 is incorrect as this reference is for South Africa and does not relate to the HDSS
b. References (7) and (13); (14) and (32); and (30) and (35) are all duplicate sets of references.**

Response: We apologise for the issues related to the incomplete list of references.

We have now carefully revised the list, amending the errors where appropriate.

Along with updating the figures to reflect the new estimates in the recently published WHO TB report (reference 1).

Once again, we appreciate the reviewers' time and effort in reviewing our work. We hope are sufficiently responsive to the critiques raised by the reviewers. Thank for considering this article for publication in PLOS Global Public Health.

Sincerely,

Dr. Alberto L. García-Basteiro, on behalf of the authors
